# How international is bioethics? A quantitative retrospective study

**DOI:** 10.1186/1472-6939-7-1

**Published:** 2006-01-13

**Authors:** Pascal Borry, Paul Schotsmans, Kris Dierickx

**Affiliations:** 1 Center for Biomedical Ethics and Law, K.U. Leuven, Kapucijnenvoer 35/3, 3000 Leuven, Belgium

## Abstract

**Background:**

Studying the contribution of individual countries to leading journals in a specific discipline can highlight which countries have the most impact on that discipline and whether a geographic bias exists. This article aims to examine the international distribution of publications in the field of bioethics.

**Methods:**

Retrospective quantitative study of nine peer reviewed journals in the field of bioethics and medical ethics (*Bioethics*, *Cambridge Quarterly of Healthcare Ethics*, *Hastings Center Report*, *Journal of Clinical Ethics*, *Journal of Medical Ethics*, *Kennedy Institute of Ethics Journal*, *Nursing Ethics*, *Christian Bioethics*, *and Theoretical Medicine and Bioethics*).

**Results:**

In total, 4,029 articles published between 1990 and 2003 were retrieved from the nine bioethical journals under study. The United States (59.3%, n = 2390), the United Kingdom (13.5%, n = 544), Canada (4%, n = 160) and Australia (3.8%, n = 154) had the highest number of publications in terms of absolute number of publications. When normalized to population size, smaller affluent countries, such as New Zealand, Finland and Sweden were more productive than the United States. The number of studies originating from the USA was decreasing in the period between 1990 and 2003.

**Conclusion:**

While a lot of peer reviewed journals in the field of bioethics profile themselves as international journals, they certainly do not live up to what one would expect from an "international" journal. The fact that English speaking countries, and to a larger extent American authors, dominate the international journals in the field of bioethics is a clear geographic bias towards the bioethical discussions that are going on in these journals.

## Background

Journals frequently lay claim to international status. The *Journal of Medical Ethics *aims to be a "leading international journal that reflects the whole field of medical ethics", the *Cambridge Quarterly of Healthcare Ethics *aims to be "an international journal that explores both broad issues in healthcare and society and organizational concerns that arise in institutions where ethics committees work", *Bioethics *is the official journal of the International Association of Bioethics that wants to be "truly international". However, there are no widely accepted standards for what it means to be international.

In many disciplines research has been executed by the curiosity to ascertain each country's contribution to the growth of scientific knowledge. Recently research has been done in the fields of anesthesiology [1], cardiology [2], and medical informatics [3]. Other geographic analyses have compared biomedical publications in the European Union and in the United States. [4,5] Studying the contribution of individual countries to leading journals in a given discipline can highlight which countries have the most impact on that discipline, and also give some idea of geographical differences between journals. As values and norms are culturally situated, it may show that international bioethics is not so international and is affected by a geographic bias.

The objective of our research is to analyse how international the discipline of bioethics is by investigating which countries contribute to international journals in the field. Therefore a study of the geographical distribution of publications in nine leading journals in the field of medical ethics and bioethics has been carried out. On the same dataset, we studied earlier authorship trends in bioethics [6], the methodology used in bioethics research [7] and the participation of developing countries to bioethics research [8].

## Methods

Our research focuses on a set of peer reviewed journals (dating from 1990 to 2003) that are explicitly dedicated to ethical issues in the context of health care and biomedicine and that are still active in 2003. The journals were selected after comparing the lists of journals indexed by Pubmed, Fangerau [9], the French 'Centre de documentation en éthique des sciences de la vie et de la santé de l'Institut National de la Santé et de la Recherche Médicale', and the German Reference Centre for Ethics in the Life Sciences. All research publications (excluding news, articles from the editors, interviews, letters, (invited) commentaries or (invited) replies to articles and cases) in peer reviewed journals indexed by the four databases were retrieved. This guaranteed that they were international journals. These journals included *Bioethics*, *Cambridge Quarterly of Healthcare Ethics*, *Hastings Center Report*, *Journal of Clinical Ethics*, *Journal of Medical Ethics*, *Kennedy Institute of Ethics Journal*, *Nursing Ethics*, *Christian Bioethics*, and *Theoretical Medicine and Bioethics *(see table 1). The two journals that were not present for the full period (1990–2003) in a Belgian library were not included in our analysis (*HEC Forum *and the *Bulletin of Medical Ethics*). Pubmed was used to obtain all electronic citations of the articles published in these journals. To verify their reliability, all journals were searched by hand and compared to the electronic dataset. Microsoft Access was used to create a template for data collection and coding. Articles were coded for the following criteria: number of authors, present occupation of author(s), countries of author(s), funding, research design, research subject, and research topic (in the case of empirical research). To guarantee reliability of data collection, the coding scheme was pilot-tested by two independent researchers. Following a discussion to resolve inconsistencies, the coding scheme was refined and then used for our review of the entire dataset. Countries were classified on the basis of the World Bank classification in high income economies and developing economies (low income and middle-income economies). The first author's affiliation was used to assign the publication's origin. The country of the institution to which the author was connected was used as a yardstick, not his personal nationality or country of origin. Population indexes (2003) were used from the World Development Indicators Database from the World Bank. Data was analysed in SAS 9.1.2 using the non-parametric chi square test for independent samples.

**Table 1:** Journals selected with year the journal was launched, publisher, location of editorial office and impact factor 2004

Journal	Year started	Publisher	Location of editorial office	Impact factor 2004
Bioethics	1987	Blackwell Publishers	United Kingdom	0.887
Cambridge quaterly of healthcare ethics	1992	Cambridge University Press	United States	/
Christian bioethics	1995	Swets & Zeitlinger	United States	/
Hastings Center Report	1971	The Hastings Center	United States	1.086
Journal of clinical ethics	1990	University Publishing Group	United States	/
Journal of medical ethics	1975	Professional and scientific publications	United Kingdom	1,353
Kennedy Institute of Ethics journal	1991	John Hopkins University Press	United States	/
Nursing Ethics	1994	Arnold	United Kingdom	0.526

Theoretical medicine and bioethics (continuation of Theoretical medicine, that was before Metamedicine)	1979	Kluwer Academic Publishers	The Netherlands	/

## Results

### Bioethics in an international context

From 1990 to 2003, 4029 articles were retrieved in the nine peer reviewed international journals under study. The articles originated from 59 different countries. The United States (59.3%, n = 2390), the United Kingdom (13.5%, n = 544), Canada (4%, n = 160) and Australia (3.8%, n = 154) had the highest number of publications in terms of absolute number of publications. These four nations account together for 80.6% of the production of articles in the bioethical journals under study. Table 2 shows the countries with 12 or more publications over the period. A further 38 countries that have published less than 12 articles each contributed only to 3.4% of the publications. When normalized to population size, smaller affluent countries, such as New Zealand, Finland and Sweden were more productive than the United States (Table 3).

**Table 2:** Number of articles from each country, percentage of total publications

Countries	Articles n	%
United States	2390	59.3
United Kingdom	544	13.5
Canada	160	4
Australia	154	3.8
Netherlands	109	2.7
Sweden	76	1.9
Germany	65	1.6
New Zealand	56	1.4
Israel	47	1.2
Finland	46	1.1
Japan	40	1
Denmark	37	0.9
Norway	27	0.7
Italy	25	0.6
China	24	0.6
Belgium	20	0.5
Turkey	18	0.4
South Africa	17	0.4
France	13	0.3
Spain	12	0.3
Switzerland	12	0.3

Rest of the World	137	3.4

**Table 3:** Number of publications in the 9 bioethical journals per million inhabitants.

	Inhabitants (thousands)	Number of articles/million inhabitants
New Zealand	4,009	14
United Kingdom	59,329	9.2
Finland	5,212	8.8
Sweden	8,956	8.5
United States	290,810	8.2
Australia	19,881	7.7
Israel	6,688	7.1
Denmark	5,387	6.9
Netherlands	16,222	6.7
Norway	4,562	5.9
Canada	31,630	5.1
Iceland	289	3.5
Ireland	3,994	2.5
Belgium	10,376	1.9
Switzerland	7,350	1.6

### Differences in journals

In 7 of the 9 analysed journals the United States are the most active contributors (Table 4). The *Journal of Clinical Ethics *(93.9%), the *Kennedy Institute of Ethics Journal *(89.6%) and the *Hastings Center Report *(89.1%) and *Christian Bioethics *(82.2%) have more than 80% of publications coming from the United States. The *Journal of Medical Ethics *and *Nursing Ethics *are the only journals with a European country that is leading the list. In both cases this is the United Kingdom. If the number of different countries is considered, the *Journal of Medical Ethics, Bioethics*, and *Nursing Ethics *are the most international journals with respectively 40, 34 and 33 different countries involved.

**Table 4:** List of the 4 countries with most publications in each journals, percentage out of the total number ot publications in each journal, the total number of countries (NC) involved in every journal

	Bioeth.	CQHE	Christ. Bioeth.	Hastings C. R.	J Clin Ethics	J Med Ethics	Ken. Institute	Nurs. Ethics	Theor. Med Bioeth
1	USA	USA	USA	USA	USA	UK	USA	UK	USA
	31.6%	76.8%	82.2%	89.1%	93.9%	43.7%	89.6%	29.7%	54.4%
2	Australia	UK	Germany	UK	Canada	USA	Canada	USA	Netherlands
	12.6%	5%	9.4%	2.2%	3%	16.5%	2%	13.4%	8.5%
3	UK	Canada	Canada	Canada	Italy	Australia	Germany	Sweden	Canada
	11.5%	3.8%	3.1%	2%	0.7%	8.8%	1.3%	8.5%	8%
4	N.Zealand	Netherlands	Greece	Netherlands	Australia	Canada	Japan	Finland	UK
	6.9%	3.2%	1.6%	1.2%	0.3%	4.4%	1.3%	6.5%	5.3%

NC	34	25	9	23	15	40	17	33	22

### U.S. contribution to bioethical journals

In table 5, we observe that studies originating from the USA are decreasing in the period between 1990 and 2003. The results of the chi-square test for two independent samples for the entire dataset indicate that the period 1997–2003 presents a significant lower number of articles orginating from the USA (χ^2 ^= 90, p < .0001) than in the period 1990–1996. While in the period 1990–1996 67.5% of the publications came from the USA, in the period 1997–2003 this had diminished to 52.7%. The introduction of *Nursing Ethics*, whose contributors are mainly European, explains only partly this tendency. The results of the chi square test without *Nursing Ethics *presents still a significant lower number of articles originating from the USA (χ^2 ^= 49.7, p < .0001) in the period 1997–2003 than in the period 1990–1996. As we described earlier [8], developing economies contributed to 3.9% (n = 156) of the publications, while high income countries contributed to 96.1% (n = 3873). The chi square test did not suggest an upward trend in publications from developing countries (χ^2 ^= 2.7, p = .10). Most publications from developing economies came from China (15.4%, n = 24), Turkey (11.5%, n = 18), South Africa (10,9%, n = 17), Hungary (7%, n = 11) and the Philippines (6.4%, n = 10). Meanwhile 51.1% (28/54) of the High Income contries indexed by the World Bank have contributed in the nine bioethical journals under study, only 20,1% (31/154) of the developing economies did so.

**Table 5:** The number of publications published from the United States of America from 1990 to 2003, in absoute numbers and in percentages

Year															Tot.
	
	1990	1991	1992	1993	1994	1995	1996	1997	1998	1999	2000	2001	2002	2003	
NOT USA	60 32.3	68 36.8	55 25.7	94 34.4	92 29.6	114 33.7	105 35	125 41.3	121 42.8	171 52.5	139 44.8	166 49.6	142 44.4	187 54.2	1639
USA	126 67.7	117 63.2	159 74.3	179 65.6	219 70.4	224 66.3	195 65	178 58.8	162 57.2	155 47.6	171 55.2	169 50.5	178 55.6	158 45.8	2390
**Total**	186	185	214	273	311	338	300	303	283	326	310	335	320	345	4029

### International research collaborations

When talking about research collaboration in the field of bioethics, it should be clear that 71.51% of all articles in our dataset are written by a single author. Two-author articles account for 16.88% (of all articles) and articles with two or more authors for 11.61%. At the level of research collaboration we observed that the group of articles with more than one author (n = 1148) has in 91.5% only 1 country of origin. In 7% (n = 81) of the cases, the contributors came from two different countries. Only exceptionally more than two countries were involved. Research collaborations were mostly combined efforts between different European countries. Only exceptionally research collaborations between U.S. and Europe or U.S. and Asia were observed.

## Discussion

In this investigation, the geographical distribution of publications in nine leading bioethical journals has been studied. Authors from 59 different countries published in these journals, but they were clearly dominated by English-speaking countries. The United States of America, the United Kingdom, Canada and Australia are the most productive countries in terms of absolute number of publications. The calculation of publications in relation to the number of inhabitants resulted in a high publication output for relatively small countries such as Finland and Sweden, but English-speaking countries as New Zealand, the UK and the USA complete the list of most productive countries. Our research reported also a low proportion of articles from authors from developing countries in the scientific literature in the field of bioethics. This corresponds with similar studies in leading medical journals [10] and many other research fields, including psychiatry [11], cardiovascular disease [12], epidemiology and HIV/AIDS [13], and tropical medicine [14]. The serious under-representation of authors from low and middle income countries in leading bioethical journals has among others to do with a language barrier, the lack of funding for research and health care, the difficult access to international journals, and editorial bias [15].

It is apparent that despite efforts to enhance cross-cultural and international perspectives on bioethics [16-18], the overwhelming majority of articles in major journals originate from a few English-speaking countries. Moreover, all editorial boards are actually in English-speaking countries. In addition, it is clear that the location of the editorial office affects the geographical origin of the contributions published. U.S. contributions are the highest in U.S. journals. Non U.S.-journals have the highest number of non U.S. contributions. While a lot of peer reviewed journals in the field of bioethics profile themselves as international journals, they certainly do not live up what one would expect from an "international" journal. Although an important reason might be that a lot of countries and regions have reputable peer-reviewed local or regional journals in their own language, the dominance of English as *lingua franca *of scientific communication offers a huge advantage to English native speakers [19]. Although most scientists recognize the benefits of a universal scientific language that enables communication with other members of the scientific communication, some have warned for the potential harmful effects of this development on other languages, on non-English speaking scholars, and on the evolution of science. [20]

The decline in the dominance of US researchers in publications in leading peer reviewed bioethical journal, is not unique to the field of bioethics. Such a trend has been observed for example in the field of anesthesiology [21], reproductive medicine [22], in clinical-research journals [23] and in surgical journals [24,25]. This relative reduction is research productivity reflects the increasing participation of Western European (mainly the United Kingdom, some Scandinavian countries and the Netherlands) countries to the field of bioethics. It is remarkable that important countries as France, Germany and Italy that belong to the G8 are only participating in a very modest way to the bioethical journals.

The fact that mainly English speaking authors participate in international bioethical journals is a clear geographic bias towards the bioethical discussions that are going on. Although we cannot assume that authors from developed (respectively developing) countries will only write about ethical problems that affect developed (respectively developing) countries, this might affect the agenda of bioethics. Turner already pointed that bioethics is biased towards ethical problems that affect wealthy developed countries and only rarely to address ethical discussions that affect developing countries such as globalisation and international inequity in access to basic goods such as food, clean water, and shelter. [26] Embryonic stem cell research, germ line therapy, and therapeutic and reproductive cloning are much more discussed in European and Western bioethics, but are only relevant for a minority of the world's population. Bioethics is certainly affected by the 10/90 gap, which is coined to point to the discripancy identified between the size of disease burden en the allocation of health research funding. 10/90 indicates that only an estimated 10% of the world's health resources are used for research into 90% of the world's health problems. [27]

Research in bioethics of course depends on substantial economic inputs. Most of the differences observed between the countries depend on the amount of funding. Nonetheless smaller initiatives may also contribute to enlarge the scope of bioethics and make bioethics more international such as the inclusion of internationally representative members in editorial boards, partnerships between high income countries and developing countries, and twinning arrangements. [28] Multilateral institutions such as the WHO and UNESCO, and particular programmes of the European Commission or the NIH can play an important role in this process.

The geographical distribution of publications in nine leading peer reviewed journals from the field of bioethics and medical ethics was investigated. Our study has, however, several limitations in the collection and interpretation of data. First, we have to remind us that publications about bioethics are not only found in journals dedicated to bioethical issues, but also in other journals such as general medical journals or speciality medical journals. Second, important journals as the *American Journal of Bioethics, the Journal of Medicine and Philosophy, Developing World Bioethics, Ethics and Medicine, Medicine Health Care and Philosophy *did not fit our selection criteria and were excluded from analysis. Although we did not intentionally limit our research to English language journals, journals as *Ethik in der Medizin *and other non English language journals could not be included for the same reason. A larger study including other bioethical journals and other medical journals should be realized to confirm the results of this study.

## Conclusion

In summary, we investigated the geographical differences in research productivity in the field of bioethics on the basis of nine leading bioethical journals for the period 1990–2003. The bioethical journals are clearly dominated by English speaking countries, and especially by the USA. Nevertheless, our study showed that the contribution of West European countries is increasing. The participation of important countries as France, Germany and Italy, however, remains very modest. Major efforts will be necessary to make bioethics a real "international" discipline, as far as this is achievable as goal.

## Competing interests

The author(s) declare that they have no competing interests.

## Authors' contributions

Pascal Borry and Kris Dierickx designed the study. After data collection by Eveline Devos and Pascal Borry, Pascal Borry performed statistical analysis. He wrote also a first draft of the article that was critically discussed by Paul Schotsmans and Kris Dierickx. Kris Dierickx coordinated the study.

## Pre-publication history

The pre-publication history for this paper can be accessed here:

http://www.biomedcentral.com/1472-6939/7/1/prepub
